# A P-Norm Robust Feature Extraction Method for Identifying Differentially Expressed Genes

**DOI:** 10.1371/journal.pone.0133124

**Published:** 2015-07-22

**Authors:** Jian Liu, Jin-Xing Liu, Ying-Lian Gao, Xiang-Zhen Kong, Xue-Song Wang, Dong Wang

**Affiliations:** 1 School of Information Science and Engineering, Qufu Normal University, Rizhao, 276826, Shandong, China; 2 Library of Qufu Normal University, Qufu Normal University, Rizhao, 276826, Shandong, China; 3 School of Information and Electrical Engineering, China University of Mining and Technology, Xuzhou, 221000, Jiangsu, China; 4 Bio-Computing Research Center, Shenzhen Graduate School, Harbin Institute of Technology, Shenzhen, 518055, Guangdong, China; 5 The Key Laboratory of Complex Systems and Intelligence Science, Institute of Automation Chinese Academy of Sciences, Beijing, 100000, China; National Institute of Plant Genome Research, INDIA

## Abstract

In current molecular biology, it becomes more and more important to identify differentially expressed genes closely correlated with a key biological process from gene expression data. In this paper, based on the Schatten *p*-norm and *L_p_*-norm, a novel *p*-norm robust feature extraction method is proposed to identify the differentially expressed genes. In our method, the Schatten *p*-norm is used as the regularization function to obtain a low-rank matrix and the *L_p_*-norm is taken as the error function to improve the robustness to outliers in the gene expression data. The results on simulation data show that our method can obtain higher identification accuracies than the competitive methods. Numerous experiments on real gene expression data sets demonstrate that our method can identify more differentially expressed genes than the others. Moreover, we confirmed that the identified genes are closely correlated with the corresponding gene expression data.

## Introduction

With the development of DNA microarray technology, it is possible for biologists to monitor the expression of thousands of genes simultaneously [1, 2]. Besides, these genes have been detected more comprehensively than ever before. A great challenge of the current bioinformatics is to explain the microarray gene expression data to gain insight into biological processes. A large number of studies have been reported to identify the characteristic genes from gene expression data. Feature extraction is a typical application of gene expression data.

A prominent feature of gene expression data is that the number of samples is far less than the number of genes. Generally speaking, on each experiment, gene expression data always contain thousands or even more than 10,000 genes, while the number of samples is generally less than 100. Statistically, it is called the small-sample-size problem, which makes many feature extraction methods lose effectiveness. The number of genes in expression data is so huge that it is quite difficult to analyze the gene expression data. Fortunately, opposed to the whole number of genes, only a small number of genes can regulate the gene expression. The minor number genes associated with a special biological process are called differentially expressed genes. Therefore, the importance of differentially expressed genes catches more and more biologists' attention. Correspondingly, it is particularly important how to discover these genes effectively.

Up to now, to find a group of genes which are relevant to a biological process from gene expression data, various feature extraction methods have been proposed for recognizing differentially expression genes. For example, Liu et al. selected characteristic genes by utilizing weight principal components by singular values [3]; the differential gene pathways were identified via principal component analysis by Ma et al. [4]; Zheng et al. selected feature genes using nonnegative matrix factorization and sparse nonnegative matrix factorization [5]. Many extraction methods, especially sparse methods, are always taking advantage of norm, and different methods using different norm. *L*_*0*_-norm and *L*_*1*_-norm are the commonly used norm, for example, for sparse principal component analysis (SPCA) method, Journée et al. took *L*_*0*_-norm penalty to analyze gene expression data [6]; for penalized matrix decomposition (PMD) method [7] which was used to extract plants differentially expressed genes responding to abiotic stress [8], *L*_*1*_-norm was taken as the penalty function. These methods have been successfully implemented on gene expression data and have high identification accuracies [9]. But the non-robust of these methods with respect to severely damaged observations in gene expression data often makes them invalid.

Recently, in the field of matrix completion, Nie et al. proposed a novel method named as joint Schatten *p*-norm and *L*_*p*_-norm robust matrix completion method for missing value recovery [10]. Matrix completion methods always presume that the values in the data matrix are associated and the rank of matrix (approximately) is low. The missing values in the data matrix can be recovered according to the observed values of the data matrix by minimizing the rank of the matrix. Therefore, the trace norm was minimized as the convex relaxation of the rank function [11–13]. Meanwhile, the prediction errors on the observed values were minimized using the squared error function by Mazumder et al.[11]. Nevertheless, the trace norm minimization may make the solution seriously deviate from the original solution in spite of it is a convex problem with a global solution. In order to solve a better approximation of the rank problem, the Schatten *p*-norm (0 ≤ *p*_*S*_ ≤ 1) is used to reformulate this problem; furthermore, the *L*_*p*_-norm (0 < *p*_*L*_ ≤ 1) is taken as the error function to improve the robustness of matrix recovery methods [10].

This method has been successfully applied to recover the data matrix in [10], however, whether the Schatten *p*-norm and *L*_*p*_-norm are effective for gene expression data analysis needs to be measured. According to [14], the gene expression data always lie near many low dimensional subspace, from which it is easy to speculate that the genes data of non-differential expression are approximately low rank. Therefore, the Schatten *p*-norm can be applied to analysis the gene expression data as well. As mentioned above, the matrix norm was widespread used to identify differentially expressed genes, so the *L*_*p*_-norm as one special form of the norm can be served as the penalty function when processing the gene expression data.

In this paper, based on the Schatten *p*-norm (0 ≤ *p*_*S*_ ≤ 1) and *L*_*p*_-norm (0 < *p*_*L*_ ≤ 1), a novel method named as *p*-norm Robust Feature Extraction (PRFE) method is put forward for identifying differentially expressed genes. In our method, we denote the gene expression data as the observed matrix **X**. To obtain the eigensamples which contain the characteristic structure of the gene expression data, matrix **X** is decomposed into **W** (the product of **U** and **D**) and **V** ^*T*^ by using SVD, where **W** is the collection of all the eigensamples [8, 15]. That is to say, the critical information of differentially expressed genes can be captured by the matrix **W**. Therefore, the optimization problem for **X** is converted into the optimization problem for **W**. We take the *L*_*p*_-norm as the error function to improve the robustness of **W**. And the Schatten *p*-norm is used as the regularization function to make **W** be a low-rank matrix which can solve the small-sample-size problem in gene expression data. Eventually, the differentially expressed genes can be identified according to the optimized **W**. The briefly introduction of PRFE is as follows: Firstly, the gene expression data matrix **X** is decomposed into two matrices **W** (the product of **U** and **D**) and **V** ^*T*^ by using SVD. Secondly, the *L*_*p*_-norm is applied to solve the optimization problem: ${\left\|W-XV\right\|}_{p_{L}}^{p_{L}}$, and the Schatten *p*-norm is used to approximate the rank of **W**: ${\left\|W\right\|}_{p_{S}}^{p_{S}}$. Thirdly, the differentially expressed genes are identified according to the optimized matrix **W**. Finally, the identified genes are appraised using the Gene Ontology tool.

To evaluate the validity of our method, both simulation data and real gene expression data sets are handled by PRFE method in the experiments. By comparing PMD and SPCA methods, all empirical results show that the novel method outperforms the competitive methods for identifying differentially expressed genes.

In summary, the main contributions of this paper are as follows:

- On one hand, based on the Schatten *p*-norm and *L*_*p*_-norm, for the first time it proposes a novel idea and method PRFE for identifying differentially expressed genes.
- On the other hand, extensive experiments are conducted on gene identification.

The remainder of the paper is structured as follows. Section 2 shows the methodology of PRFE. Then how to identify differentially expressed genes using PRFE is introduced. The experimental results on simulation data and real gene expression data sets are presented in Section 3. In Section 4, the conclusion is shown.

## Methodology

### 2.1 Definitions of *Lp*-norm and Schatten *p*-norm

For a matrix **W** contains *m* rows and *n* columns, the *L*_*p*_-norm (0 < *p*_*L*_ < ∞) to the power *p*_*L*_ can be defined as

$${\left\|W\right\|}_{p_{L}}^{p_{L}}=\sum_{i}^{m}\sum_{j}^{n}{\left|w_{ij}\right|}^{p_{L}},$$ (1)

where *w*_*ij*_ is the *i*-th row and *j*-th column element of **W**.

The extended Schatten *p*-norm (0 < *p*_*S*_ < ∞) of the matrix **W** to the power *p*_*S*_ can be written as

$${\left\|W\right\|}_{p_{S}}^{p_{S}}=\sum_{i=1}^{\text{min}\left\{m,n\right\}}\sigma_{i}^{p_{S}},$$ (2)

where *σ*_*i*_ is the *i*-th singular value of **W**. When *p*_*S*_ = 1, the Schatten 1-norm is also known as the nuclear norm or trace norm, which is usually taken as the following form: ‖**W**‖_*_. When *p*_*S*_ = 0, if we define 0^0^ = 0, Eq 2 is the rank of **W** [10].

### 2.2 The definition of PRFE

Denote by **X** an *m*×*n* matrix, each row of **X** represents the expression level of a gene in *n* samples, and each column of **X** represents the expression level of all the *m* genes in one sample. As mentioned above, for gene expression researches, the gene number *m* is much larger than the sample number *n*. The PRFE method decomposes the matrix **X** into two matrices **W** (the product of **U** and **D**) and **V** ^*T*^ by using SVD

$$X∼WV^{T},$$ (3)

where **W** is an *m*×*K* matrix and **V** ^*T*^ is a *K*×*n* matrix, **VV** ^*T*^ = **I**_*n*_. The general feature extraction minimization problem [7, 8] is defined as follows:

$$\underset{X}{\text{min}}{\left\|X-WV^{T}\right\|}_{F}^{2},$$ (4)

where ‖•‖_*F*_ is the Frobenius norm. The differentially expressed genes are usually identified according to **W** [8, 15], so the Eq 4 can be easily converted to the following form:

$$\underset{W}{\text{min}}{\left\|XV-WV^{T}V\right\|}_{F}^{2}=\underset{W}{\text{min}}{\left\|W-XV\right\|}_{F}^{2},$$ (5)

which can make it more convenient to optimize **W**. To improve the robustness to outliers in gene expression data, we use the *L*_*p*_-norm (0 < *p*_*L*_ ≤ 1) to obtain an optimized **W**:

$$\underset{W}{\text{min}}{\left\|W-XV\right\|}_{p_{L}}^{p_{L}}.$$ (6)

When *p*_*S*_ → 0, relative to the trace norm ‖**W**‖_*_, Schatten *p*-norm ${\left\|W\right\|}_{p_{S}}^{p_{S}}$ will approximate the rank of **W** [16], hence, we replace the ‖**W**‖_*_ by Schatten *p*-norm (0 ≤ *p*_*S*_ ≤ 1) ${\left\|W\right\|}_{p_{S}}^{p_{S}}$. Finally, the PRFE method can be used to solve the feature extraction problem as follows:

$$\underset{W}{\text{min}}{\left\|W-XV\right\|}_{p_{L}}^{p_{L}}+\lambda {\left\|W\right\|}_{p_{S}}^{p_{S}},$$ (7)

where *λ* is the regularization parameter.

### 2.3 Solving the PRFE problem

Eq 7 is intractable since the two items are non smooth. Therefore, the Augmented Largrangian Multiplier (AML) method [17–19] is taken to solve Eq 7. In this subsection, we first introduce the AML method briefly.

For a matrix **A**, the constrained optimization problem can be written as

$$\underset{A}{\text{min}}\;f(A).$$ (8)

Suppose that the matrix **B** satisfies the condition that **B** = **A**, then the AML algorithm to solve Eq 8 is described as follows:

**Algorithm 1.** AML algorithm to solve Eq 8

Set 1 < *η* < 2. Initialize **Ω** and *φ* > 0.

while not converge do

Update **A** by $\underset{A}{\text{min}}\;f(A)+\frac{\varphi }{2}{\left\|B-A+\frac{1}{\varphi }\Omega \right\|}_{F}^{2}$

Update **Ω** by **Ω** = **Ω** + **B** − **A**

Update *φ* by *φ* = *ηφ*

end while

To facilitate the writing, in Eq 7 we replace the **W** − **XV** with **C** and replace **W** with **D**. According to AML algorithm, Eq 7 can be rewritten as follows:

$$\begin{array}{l} \underset{W,A,B}{\text{min}}{\left\|C\right\|}_{p_{L}}^{p_{L}}+\lambda {\left\|D\right\|}_{p_{S}}^{p_{S}}+\frac{\varphi }{2}{\left\|C-W+XV+\frac{1}{\varphi }\Omega \right\|}_{F}^{2}\\ \;+\frac{\varphi }{2}{\left\|W-D+\frac{1}{\varphi }\Psi \right\|}_{F}^{2}. \end{array}$$ (9)

In Eq 9, there are three variables **W**, **C** and **D** which make the formula quite difficult to be solved. The alternating direction method [20] can be utilized to deal with this thorny problem exactly. The core idea to resolve Eq 9 is the case that the problem is optimized only by one variable when fixing the remaining two variables. In this way, three new but solvable problems arise.

**Problem 1**: When fixing **W** and **D**, Eq 9 can be written as the following form:

$$\underset{C}{\text{min}}{\left\|C\right\|}_{p_{L}}^{p_{L}}+\frac{\varphi }{2}{\left\|C-W+XV+\frac{1}{\varphi }\Omega \right\|}_{F}^{2}.$$ (10)

In this case, $W-XV-\frac{1}{\varphi }\Omega$ can be denote as a constant *e*. And note that the elements in **W** can be decoupled, so for each element, only the following problem need to be solved:

$$\underset{w}{\text{min}}\frac{1}{2}{\left(w-e\right)}^{2}+\tau {\left|w\right|}^{p_{L}},$$ (11)

where *τ* denotes $\frac{1}{\varphi }$. Then we denote *f*(*w*) as the objective function in Eq 11:

$$f(w)=\frac{1}{2}{\left(w-e\right)}^{2}+\tau {\left|w\right|}^{p_{L}}.$$ (12)

In Eq 12, there is only one variable *w*, and the convexity of the equation can be easily analyzed. When *w* = 0, *f*(*w*) is not differentiable, so we only consider the case of *w* ≠ 0 in the following analysis. Then we compare the minimal solution to *f*(*w*) (*w* ≠ 0) with $f(0)=\frac{1}{2}e^{2}$ to obtain the optimum solution to *f*(*w*). When *w* ≠ 0, the first, second and third derivatives of *f*(*w*) are as follows:

$$f\prime (w)=w-e+\tau p_{L}{\left|w\right|}^{p_{L}-1}\;\text{sgn}(w),$$ (13)

$$f\prime \prime (w)=1-\tau p_{L}(1-p_{L}){\left|w\right|}^{p_{L}-2},$$ (14)

$$f\prime \prime \prime (w)=\tau p_{L}(1-p_{L})(2-p_{L}){\left|w\right|}^{p_{L}-3}\;\text{sgn}(w),$$ (15)

where sgn(*w*) is defined as follows: sgn(*w*) = 1 if *w* > 0, and sgn(*w*) = −1 if *w* < 0. The local minimum of *f*(*w*) can be obtained by finding the root of *f*′(*w*) = 0, so we analysis *f*′(*w*) at first. According to Eq 15, *f*′(*w*) is convex at *w* > 0 and *f*′(*w*) is concave at *w* < 0. In order to find the extrema of *f*′(*w*), we let *f*″(*w*) = 0 and obtain the solution:

$$\left|w\right|={\left(\tau p_{L}(1-p_{L})\right)}^{\frac{1}{2-p_{L}}}.$$ (16)

In this case, we denote a constant *a* (*a* > 0) as *w* (*w* > 0), that is *f*″(*a*) = 0 and *f*″(−*a*) = 0. Therefore, *f*′(*w*) can obtain the maximum *f*″(−*a*) at *w* < 0, and *f*′(*w*) can obtain the minimum *f*″(*a*) at *w* > 0. There are three cases to solve *f*(*w*):

(a) *f*′(*a*) ≥ 0 and *f*′(−*a*) ≤ 0

In this case, *f*′(*w*) ≤ 0 when *w* < 0 and *f*′(*w*) ≥ 0 when *w* > 0, so the minimal solution to *f*(*w*) is *w* = 0.

(b) *f*′(−*a*) > 0

In this case, according to Eq 13, *f*′(*a*) > 0. *f*′(*w*) ≥ 0 when *w* > 0 and *w* < 0, *f*′(*w*) = 0 has two roots which indicate that *f*(*w*) is convex at *w* < −*a* and *f*(*w*) is concave at –*a* ≤ *w* < 0. So the minimal solution to *f*(*w*) is the root of *f*′(*w*) = 0 at *e* < *w* < −*a*.

(c) *f*′(*a*) < 0

In this case, according to Eq 13, *f*′(−*a*) < 0. *f*′(*w*) ≤ 0 when *w* < 0 and *w* > 0, *f*′(*w*) = 0 has two roots which indicate that *f*(*w*) is convex at *w* > *a* and *f*(*w*) is concave at 0 ≤ *w* < *a*. So the minimal solution to *f*(*w*) is the root of *f*′(*w*) = 0 at *a* < *w* < *e*.

In summary, the Eq 11 can be optimized by

$$\left\{ \begin{array}{l} f\prime (a)\geq 0\;\text{and}\;f\prime (-a)\leq 0,\;w=0\\ f\prime (-a)>0\;\text{and}\;f\prime (a)>0,\;w=\text{arg}\;\underset{w\in \left\{0,w_{1}\right\}}{\text{min}}\;f(w)\\ f\prime (a)<0\;\text{and}\;f\prime (-a)<0,\;w=\text{arg}\;\underset{w\in \left\{0,w_{2}\right\}}{\text{min}}\;f(w), \end{array}\right.$$ (17)

where *w*_1_ ∈ (*e*, −*a*) and *w*_2_ ∈ (*a*, *e*) are the roots of *f*′(*w*) = 0. The roots can be acquired by the Newton method initialized at *e* [10].

**Problem 2**: When fixing **W** and **C**, Eq 9 can be written as:

$$\underset{D}{\text{min}}\;\lambda {\left\|D\right\|}_{p_{S}}^{p_{S}}+\frac{\varphi }{2}{\left\|W-D+\frac{1}{\varphi }\Psi \right\|}_{F}^{2}.$$ (18)

In this case, $W+\frac{1}{\varphi }\Psi$ can be denoted as **E**. For simplicity, Eq 18 can be rewritten as follows:

$$\underset{D}{\text{min}}\frac{1}{2}{\left\|D-E\right\|}_{F}^{2}+\rho {\left\|D\right\|}_{p_{S}}^{p_{S}},$$ (19)

where *ρ* denotes $\frac{\lambda }{\varphi }$. Suppose **D** and **E** are decomposed into **UΔV** ^*T*^ and **QΣR** ^*T*^, respectively, where **Δ** and **Σ** are the singular value matrices. So Eq 19 can be written as

$$\underset{D}{\text{min}}\frac{1}{2}{\left\|U\Delta V^{T}-Q\Sigma R^{T}\right\|}_{F}^{2}+\rho {\left\|\Delta \right\|}_{p_{S}}^{p_{S}}.$$ (20)

To obtain the solution of Eq 20, we first introduce the theorem: For any two matrices $A,B\in {\mathbb{R}}^{m\times n}$, then *tr*(**A** ^*T*^ **B**) ≤ *tr*(*σ*(**A**)^*T*^ *σ*(**B**)), where *σ*(**A**) and *σ*(**B**) are the singular value matrices of **A** and **B**, respectively. According to the theorem, we have the following formula

$${\left\|U\Delta V^{T}-Q\Sigma R^{T}\right\|}_{F}^{2}\geq {\left\|\Delta -\Sigma \right\|}_{F}^{2}.$$ (21)

When **U** = **Q** and **V** ^*T*^ = **R** ^*T*^, the equality holds in Eq 21, so the optimal problem in Eq 20 can be converted as the following form

$$\underset{\Delta }{\text{min}}\frac{1}{2}{\left\|\Delta -\Sigma \right\|}_{F}^{2}+\rho {\left\|\Delta \right\|}_{p_{S}}^{p_{S}}.$$ (22)

Suppose *σ*_*i*_ and *δ*_*i*_ are the *i*-th singular values of **D** and **E**, respectively, then Eq 22 can be written as

$$\underset{\sigma_{{}^{i}}}{\text{min}}\frac{1}{2}{\sum_{i}\left(\sigma_{i}-\delta_{i}\right)}^{2}+\rho \sum_{i}\sigma_{{}_{i}}^{p_{S}}.$$ (23)

The form of Eq 23 is the same as Eq 11, so the optimal solution to Eq 23 can be obtained in the same way with the optimal solution of Eq 11.

**Problem 3:** When fixing **C** and **D**, Eq 9 can be written as:

$$\underset{W}{\text{min}}\frac{\varphi }{2}{\left\|C-W+XV+\frac{1}{\varphi }\Omega \right\|}_{F}^{2}+\frac{\varphi }{2}{\left\|W-D+\frac{1}{\varphi }\Psi \right\|}_{F}^{2}.$$ (24)

Denote $F=C+XV+\frac{1}{\varphi }\Omega$ and $G=D-\frac{1}{\varphi }\Psi$, Eq 24 can be simplified to the following form:

$$\underset{W}{\text{min}}\frac{\varphi }{2}{\left\|W-F\right\|}_{F}^{2}+\frac{\varphi }{2}{\left\|W-G\right\|}_{F}^{2}.$$ (25)

The problem in Eq 25 is equivalent to solving a quadratic function and it is easy to obtain the solution

$$W=\frac{F+G}{2}.$$ (26)

In summary, the brief algorithm of PRFE is shown as follows.

**Algorithm 2.** PRFE method

Input: Data matrix: $X\in {\mathbb{R}}^{m\times n}$

Schatten *p*-norm value: *p*_*S*_

*L*_*p*_-norm value: *p*_*L*_

Regularization parameter: *λ*

Output: Optimized matrix $W\in {\mathbb{R}}^{m\times n}$

The data matrix **X** is decomposed into **W** and **V** ^*T*^ by SVD, where **W** is the product of **U** and **D**.

**W** ⇐ **XV**.

Solve the problem in Eq 7 by AML method.

Set 1 < *η* < 2. Initialize **C**, **D**, **Ω**, **Ψ** and *φ* > 0.

while not converge do

Update **C** by the optimal solution to Eq 10

Update **D** by the optimal solution to Eq 18

Update **W** by Eq 26

Update **Ω** by **Ω** = **Ω** + *φ*(**C** – **W** + **XV**)

Update **Ψ** by **Ψ** = **Ψ** + *φ*(**W** − **D**)

Update *φ* by *φ* = *ηφ*

end while

### 2.4 Identifying differentially expressed genes by PRFE

**Fig 1:**
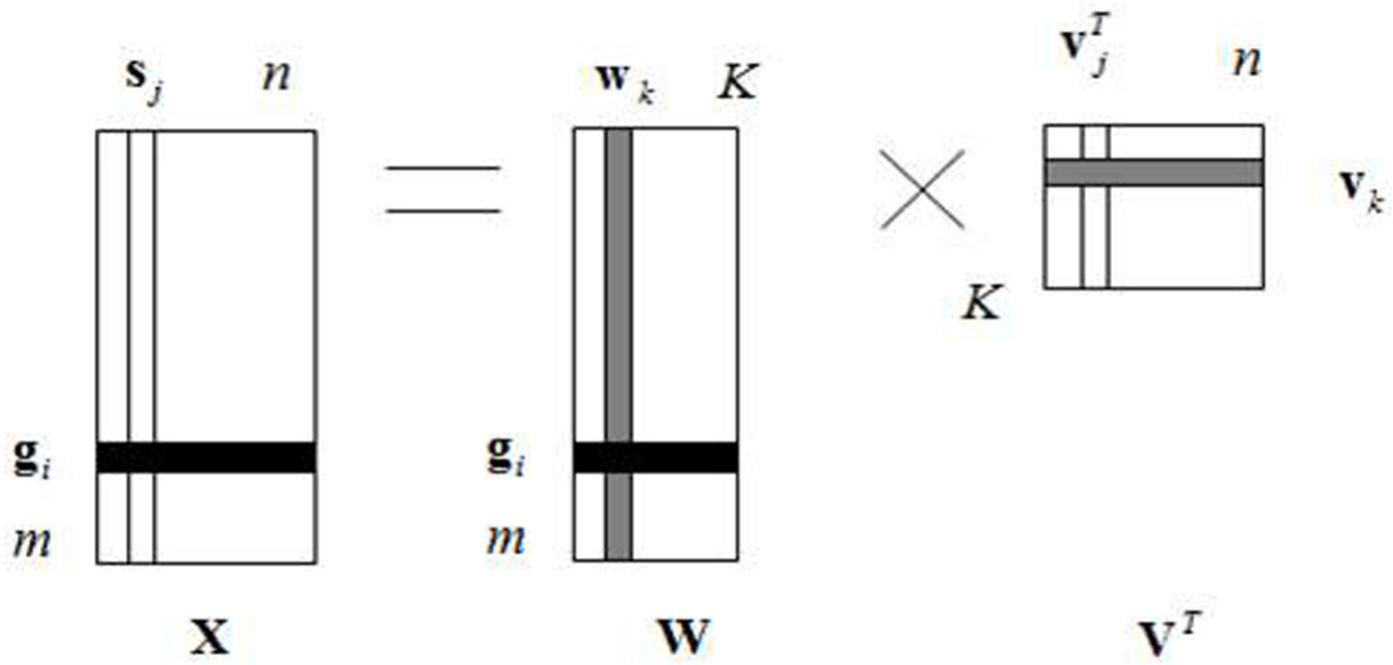
The PRFE model of gene expression data used for gene identification.

The gene expression data can be denoted as a matrix **X** of size *m*×*n*, each row of **X** represents the expression level of a gene in *n* samples, and each column of **X** represents the expression level of all the *m* genes in one sample. Fig 1 shows the graphical depiction of processing the matrix **X** by PRFE. Following the convention in [15], the PRFE method decomposes the matrix **X** into two matrices **W** and **V** ^*T*^, where **s**_*j*_ (*j* = 1,2,⋯, *n*) is the sample expression profile, **g**_*i*_ is the gene transcriptional responses, **w**_*k*_ is an eigensample of column of **W**, **v**_*k*_ is an engienpattern of row of **V** ^*T*^, $v_{j}^{T}$ is the *j*-th column of **V** ^*T*^ and contains the coordinates of the *j*-th sample in **X**.

To identify differentially expressed genes from **X**, the critical information of the differentially expressed genes in **s**_*j*_ needs to be studied. Since $v_{j}^{T}$ includes the positional information of the *j*-th sample in **X**, according to the formula

$$s_{j}=\sum_{k=1}^{K}w_{k}v_{jk},\;j=1,2,\cdots n,$$ (27)

the important information of differentially expressed genes in **s**_*j*_ can be captured by **w**_*k*_. That is, the differentially expressed genes are identified based on **W**.

After **W** has been optimized by PRFE method, a novel $\tilde{W}$ can be obtained. Therefore, according to $\tilde{W}$, the differentially expressed genes are identified. $\tilde{W}$ can be denoted as follows:

$$\tilde{W}=\left[\begin{array}{cccc} {\tilde{w}}_{11} & {\tilde{w}}_{12} & \cdots & {\tilde{w}}_{1n}\\ {\tilde{w}}_{21} & {\tilde{w}}_{22} & \cdots & {\tilde{w}}_{2n}\\ \vdots & \vdots & \ddots & \vdots \\ {\tilde{w}}_{m1} & {\tilde{w}}_{m2} & \cdots & {\tilde{w}}_{mn} \end{array}\right].$$ (28)

Following the description in [21], the differentially expressed genes are usually grouped into two classes: up-regulated genes and down-regulated genes, which can be reflected by the positive items and negative items in $\tilde{W}$. Here, we only consider the absolute value of the items in $\tilde{W}$ to identify the differentially expressed genes. Then, the matrix is summed by rows to obtain the evaluating vector $\overset{\wedge }{W}$ [22]

$$\overset{\wedge }{W}={\left[\sum_{j=1}^{n}\left|{\tilde{w}}_{1j}\right|\;\sum_{j=1}^{n}\left|{\tilde{w}}_{2j}\right|\;\cdots \;\sum_{j=1}^{n}\left|{\tilde{w}}_{mj}\right|\right]}^{T}.$$ (29)

Generally speaking, the larger the item in $\overset{\wedge }{W}$ is, the more differential the gene is. Therefore, we sort the elements in $\overset{\wedge }{W}$ in descending order and take the top *h* (*h* ≪ *m* is a number that can be defined according to the corresponding requirement) genes as the differentially expressed ones.

### 2.5 Discussion of the selection of *p* value in PRFE

In Eq 7, the values of *p*_*L*_ and *p*_*S*_ in PRFE method are specified within 0 < *p*_*L*_ ≤ 1 and 0 ≤ *p*_*S*_ ≤ 1, respectively. However, the special values of *p*_*L*_ and *p*_*S*_ are more interesting to be selected for solving the problem in Eq 7.

To improve the robustness to outliers in gene expression data, the *L*_*p*_-norm is taken as the error function. In PRFE, the value of *p*_*L*_ should be in the range of (0, 1], and it does not mean that the smaller value of *p*_*L*_ can acquire the better performance. Conversely, we suggest taking the *L*_*1*_-norm to improve the robustness to outliers since the error function is convex while *p*_*L*_ = 1 [10].

The Schatten *p*-norm is used as the regularization function to obtain a low-rank matrix. As mentioned in Subsection 2.1 and 2.2, when *p*_*S*_ → 0, the Schatten *p*-norm approximates the rank function. That is, the Schatten *p*-norm can achieve more accurate to approximate rank in the range of [0, 1). However, in this case the Schatten *p*-norm is not convex. When *p*_*S*_ = 1, the Schatten *p*-norm is convex while it cannot achieve accurate to approximate rank [10]. Thus, we have the flexibility to choose different *p*_*S*_ values corresponding to the different situations.

In order to verify the reasonableness of the values of *p*_*L*_ and *p*_*S*_, the simulation experiments are given in Subsection 3.1.

## Results and Discussion

This section shows the experimental results on simulation data and real gene expression data sets. For simplicity, the regularization parameter *λ* in Eq 7 is taken as 1 in whole experiments [10]. To demonstrate the effectiveness of our method for recognizing the differentially expressed genes, the PMD [7], SPCA [6], CIPMD [9] and SVM-RFE [23]are used for comparison.

### 3.1 Results on simulation data

#### 3.1.1 Data source

We describe here a general scheme to generate simulation data. Suppose we want to generate data from ${\mathbb{R}}^{p}$ such that the *q* (*q* < *p*) leading eigenvectors of the covariance matrix **Σ** are sparse. Denote the first *q* eigenvectors as **v**_1_,⋯, **v**_*q*_, which are specified to be sparse and orthonormal. The remaining *p* − *q* eigenvectors are not specified to be sparse. Denote the positive eigenvalues of **Σ** in decreasing order as *c*_1_,⋯,*c*_*p*_.

We first need to generate the other *p* − *q* orthonormal eigenvectors of **Σ**. To this end, form a full-rank matrix $V^{*}=[v_{1},\cdots ,v_{q},v_{q+1}^{*},\cdots ,v_{p}^{*}]$, where **v**_1_,⋯,**v**_*q*_ are the pre-specified sparse eigenvectors and $v_{q+1}^{*},\cdots ,v_{p}^{*}$ are arbitrary. For example, $v_{q+1}^{*},\cdots ,v_{p}^{*}$ can be randomly drawn from (0, 1); if **V** ^*^ is not of full-rank for one random draw, we can draw another set of vectors. Then we apply the Gram-Schmidt orthogonalization method to **V** ^*^ to obtain an orthogonal matrix **V** = [**v**_1_,⋯ **v**_*q*_, **v**_*q*+1_,⋯, **v**_*p*_], which is actually the matrix **Q** from the QR decomposition of **V** ^*^. Given the orthogonal matrix **V**, we form the covariance matrix **Σ** using the following eigen decomposition expression $\Sigma =c_{1}v_{1}v_{1}^{T}+c_{2}v_{2}v_{2}^{T}+\cdots +c_{p}v_{p}v_{p}^{T}=VCV^{T}$, where **C** = diag{*c*_1_,⋯, *c*_*p*_} is the eigenvalue matrix. The first *q* eigenvectors of **Σ** are the pre-specified sparse vectors **v**_1_,⋯, **v**_*q*_. To generate data from the covariance matrix **Σ**, let **Σ** be a random draw from *N*(0, *I*_*p*_) and X=VC12Z, then cov(**X**) = **Σ**, as described in [24].

The simulation data are generated as **X** ∼ (**0,** ∑_4_) with *m* = 3000. Let ${\tilde{v}}_{1}∼{\tilde{v}}_{4}$ be four 3000-dimensional vectors, such as ${\tilde{v}}_{1k}=1,\;k=1,\cdots ,125$, and ${\tilde{v}}_{1k}=0,\;k=126,\cdots ,3000$; ${\tilde{v}}_{2k}=1,\;k=126,\cdots ,250$, and ${\tilde{v}}_{2k}=0,\;k\neq 126,\cdots ,250$; ${\tilde{v}}_{3k}=1,\;k=251,\cdots ,375$, and ${\tilde{v}}_{3k}=0,\;k\neq 251,\cdots ,375$; ${\tilde{v}}_{4k}=1,\;k=376,\cdots ,500$, and ${\tilde{v}}_{4k}=0,\;k\neq 376,\cdots ,500$. Let **E** ∼ *N*(0, 1) be a noise matrix with 3000-dimension, which is added into $\tilde{v}$. The four eigenvectors of ∑_4_ can be denoted as $v_{k}={\tilde{v}}_{k}/\left\|{\tilde{v}}_{k}\right\|,\;k=1,2,3,4$. And to make the four eigenvectors dominate, the eigenvalues in **X** can be denoted as *c*_1_ = 200, *c*_2_ = 150, *c*_3_ = 100, *c*_4_ = 50 and *c*_*k*_ = 1 for *k* = 5,⋯, 3000. The detailed synthetic idea can be found in [24].

#### 3.1.2 Simulation results

**Fig 2:**
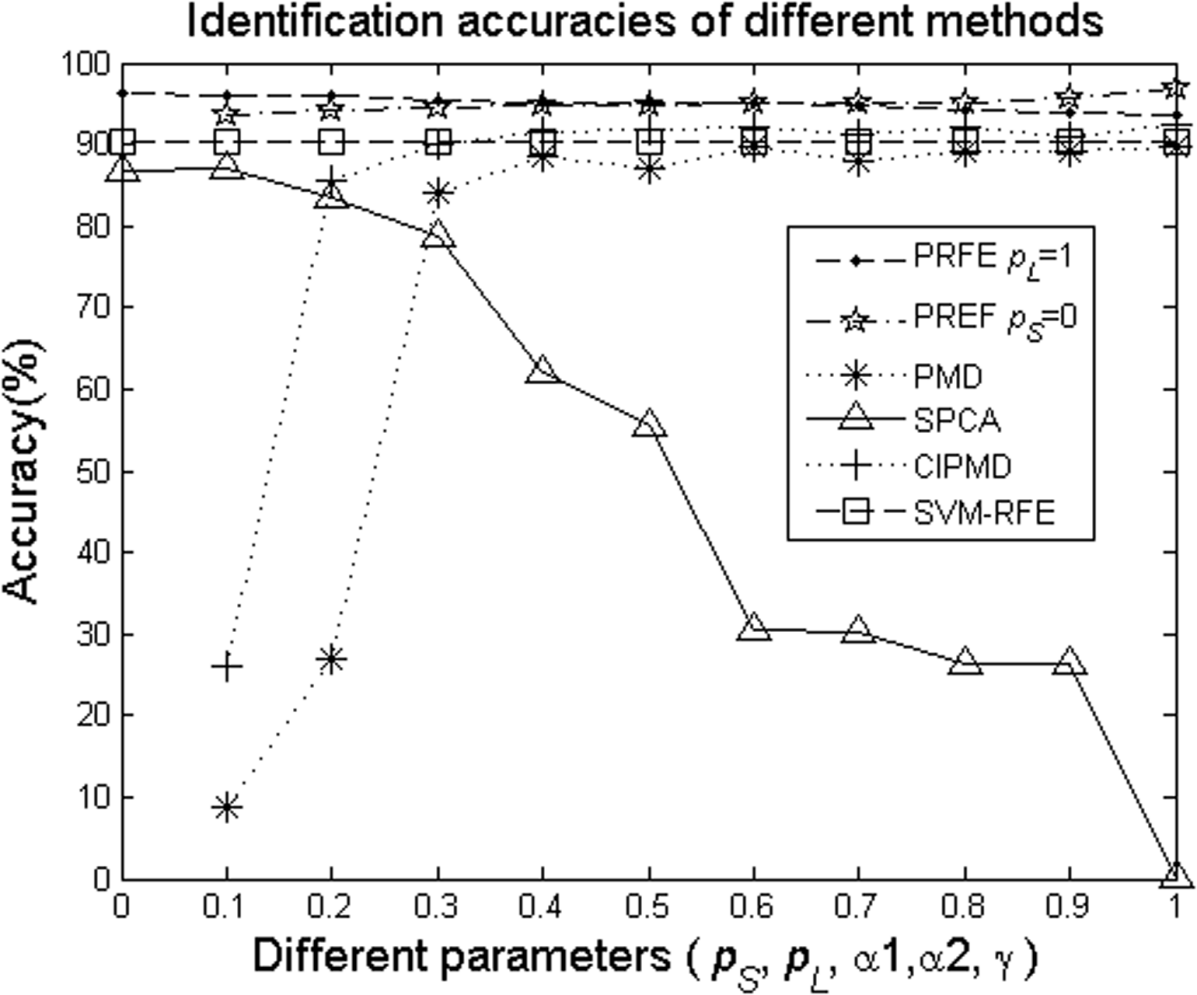
Identification accuracies of the five methods on simulation data with different parameters, where *p*_*S*_ is taken as the parameter in the case of PRFE *p*_*L*_ = 1 to test the performance of different *p*_*S*_ values; *p*_*L*_ is taken as the parameter in the case of PRFE *p*_*S*_ = 1 to test the performance of different *p*_*L*_ values; *α*_1_, *α*_2_ and *γ* are the control-sparsity parameters of PMD, CIPMD and SPCA, respectively.

In order to evaluate the performance of five methods, the experiment is repeated for 30 times and the average identification accuracies are reported. For fair comparison, 500 genes are identified by the five methods with their unique parameters. Since PMD, CIPMD and SPCA are sparse methods, *α*_1_, *α*_2_ and *γ* are the control-sparsity parameters of PMD, CIPMD and SPCA, respectively. Because *p*_*L*_ and *p*_*S*_ are the most important parameters of PRFE method, their impacts on our method should be studied at first. According to Subsection 2.5, we suggest taking *p*_*L*_ = 1 to improve the robustness to outliers and taking *p*_*S*_ = 0 to approximate the rank function. Therefore, when *p*_*L*_ = 1, we test the performance of our model with different values of *p*_*S*_ in the range of {0, 0.1,⋯, 1} and define this special case as PRFE *p*_*L*_ = 1. Similarly, when *p*_*S*_ = 0, we investigate the performance of our method with different values of *p*_*L*_ in the range of {0.1, 0.2,⋯, 1} and define this special case as PRFE *p*_*S*_ = 0. Fig 2 shows the average identification accuracies of the five methods with different parameters while the simulation data are 3000×10. In Fig 2, it can be clearly seen that either PRFE *p*_*L*_ = 1 or PRFE *p*_*S*_ = 0 is superior to the other four methods in spite of PMD, SPCA, CIPMD and SVM-RFE can also reach higher identification accuracies. This result clearly justifies the serviceability of the PRFE method to introduce *p*_*L*_-norm and *p*_*S*_-norm in gene identification. To be precise, while the parameters are larger than 0.4, PMD and CIPMD reach their highest point and becomes stable. The accuracies of SPCA is monotonically decreasing when the parameter are larger than 0.1. Due to SVM-RFE is not a sparse method, so it is not sensitive to the parameters. The accuracies of PRFE *p*_*L*_ = 1 is also monotonically decreasing in all of the parameters, this verifies the Schatten *p*-norm can achieve more accurate to approximate rank when *p*_*S*_ → 0. The accuracies of PRFE *p*_*S*_ = 0 is increasing with the increasing parameters which can demonstrate that *L*_*p*_-norm, as the error function, can acquire a better performance when *p*_*L*_ = 1.

**Fig 3:**
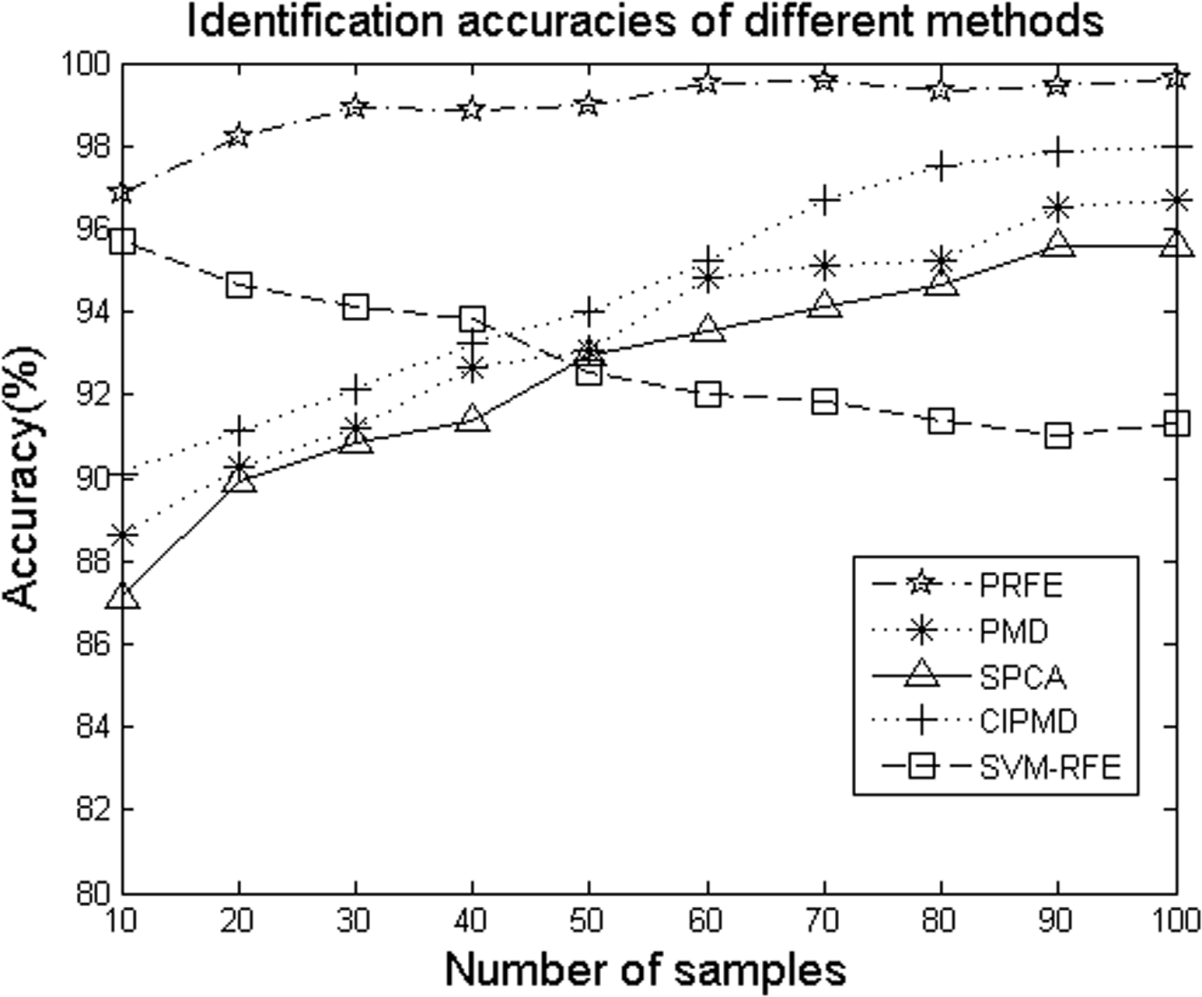
Identification accuracies of the five methods on simulation data with different samples.

The number of samples in gene expression data has an influence on the identification accuracy when we recognize differentially expressed genes using feature extraction methods. The five methods are tested with different sample numbers to find the regular pattern that how the sample numbers affect the identification accuracy. Here, for the PRFE method, we select *p*_*L*_ = 1 and *p*_*S*_ = 0 since PRFE can obtain the best result in this case. PMD and CIPMD can reach its highest point and becomes stable when the parameters are larger than 0.4, so we select 0.4 as the sparse parameter for PMD and CIPMD. For SPCA, we choose 0.1 as the sparse parameter since SPCA can acquire its best result when parameter is 0.1. Fig 3 shows the average identification accuracies of different methods with different sample numbers. It is obvious to be seen that with the increasing of sample numbers, the accuracies of the four methods are increased. The accuracies of SVM-RFE method is monotonically decreasing. The proposed method can dominate the other methods in all the sample numbers. Moreover, the accuracies of our method are close to 100% when *n* ≥ 60.

**Fig 4:**
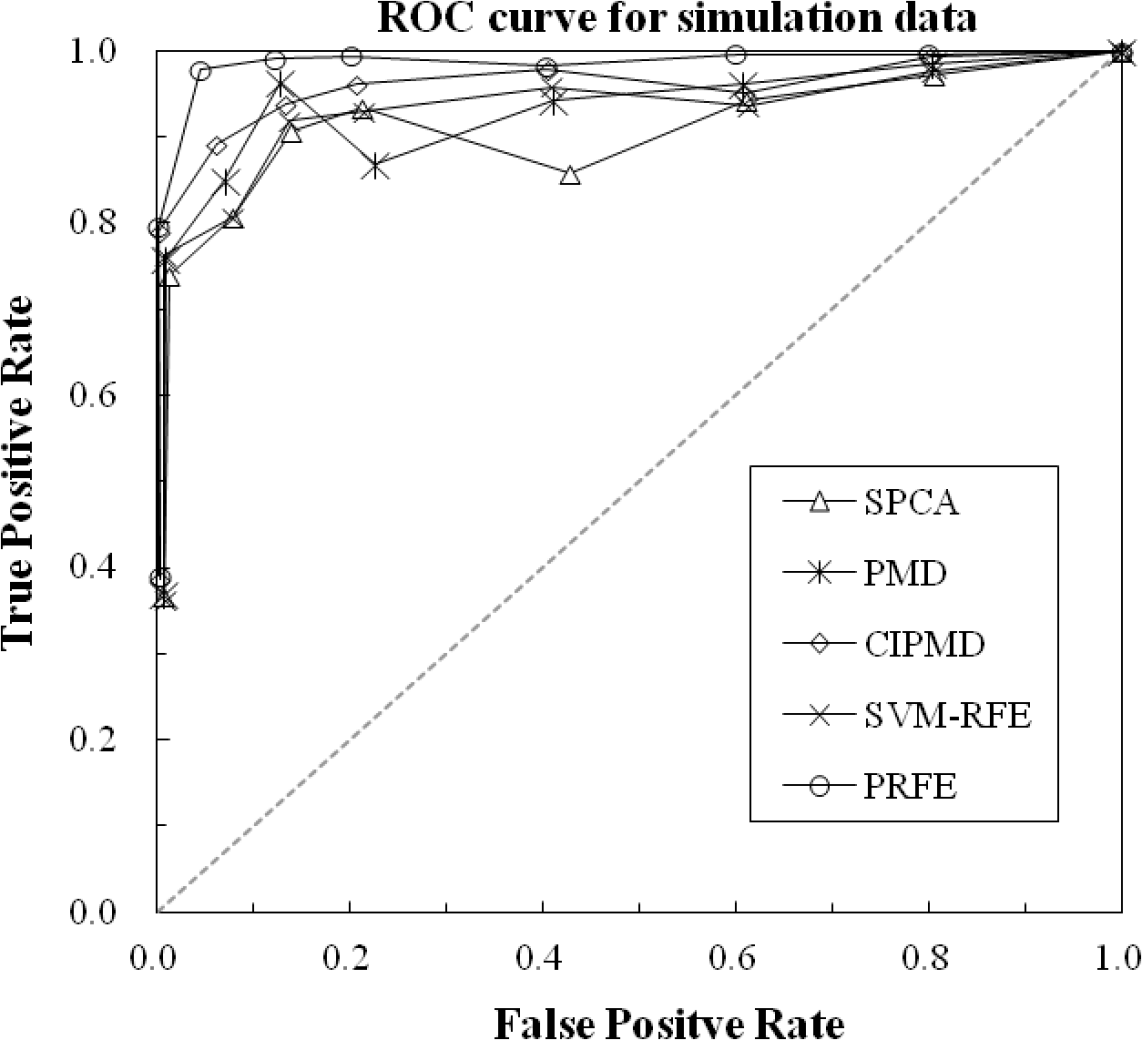
ROC curve for simulation data.

To further investigate the performance of the methods, the average receiver operator characteristic (ROC) curve is shown in Fig 4 with the optimal parameter of different methods. Fig 4 shows that PRFE and the competitive methods can identify differentially expressed genes effectively. However, through the True Positive Rate and False Positive Rate we can find that PRFE have the best outcome. Since we add a noise matrix into simulation data, so the false positive and false negative appear.

The area under curve (AUC) statistics are listed in Table 1 with the optimal parameter of different methods. From Table 1 we can conclude that the ascending order of accuracy given by the five methods is: SPCA, PMD, SVM-RFE, CIPMD and PRFE.

**Table 1:** AUC statistics for simulation data.

Methods	SPCA	PMD	CIPMD	SVM-RFE	PRFE
**AUC**	0.909	0.911	0.959	0.933	0.990

### 3.2 Results on gene expression data sets

To evaluate the proposed method, two publically available gene expression data are adopted: gene expression data of plants responding to abiotic stresses [25, 26] and the leukemia data set [27]. To compare with PRFE method, PMD, CIPMD, SVM-RFE and SPCA are also used to identify differentially expressed genes.

#### 3.2.1 Parameters selection

As mentioned in Subsection 3.1, PRFE method can reach the best performance when *p*_*L*_ = 1 and *p*_*S*_ = 0. Therefore, for PRFE method, we take *p*_*L*_ = 1 and *p*_*S*_ = 0 to identify the differentially expressed genes on real gene expression data. PMD, CIPMD and SPCA are parse methods, whose sparse parameters have an enormous influence on the identification accuracy. According to the results on simulation data in Subsection 3.1, by choosing the sparse parameters *α*_1_, *α*_2_ and *γ* appropriately, PMD, CIPMD and SPCA can obtain their optimal performance respectively.

#### 3.2.2 Results on gene expression data of plants responding to abiotic stresses

(a) Data source and GO analysis

Gene expression data of plants responding to abiotic stresses include two classes: roots and shoots in each stress. The Affymetix CEL files were downloaded from NASCArrays [http://affy.arabidopsis.info/link_to_iplant.shtml] [25], reference numbers are: control, NASCArrays-137; cold stress, NASCArrays-138; osmotic stress, NASCArrays-139; salt stress, NASCArrays-140; drought stress, NASCArrays-141; UV-B light stress, NASCArrays-144; and heat stress, NASCArrays-146. There are 22810 genes in each sample and the sample number of each stress type in the raw data is listed in Table 2. The raw arrays are adjusted by using GC-RMA software [26] in order to avoid the background of optional noise and normalized by using quantile normalization. The GC-RMA results are gathered in a matrix to be processed by SPCA, PMD, CIPMD, SVM-RFE and PRFE.

**Table 2:** The sample number of each stress type in the raw data.

Stress Type	control	cold	drought	heat	osmotic	salt	UV-B
Sample Number	8	6	7	8	6	6	7

For fair comparison, 500 genes are identified by PMD, CIPMD and SPCA by choosing *α*_1_, *α*_2_ and *γ* appropriately. SVM-RFE has no sparse parameters, the top 500 genes of SVM-RFE method are selected as the differentially expressed genes. And according to Subsection 2.4, the top 500 genes of PRFE method are selected as the differentially expressed genes. The identified genes are checked by Gene Ontology (GO) Term Enrichment tools which can be used to describe genes in the input or query set and to help discover what functions the genes may have in common [28]. GOTermFinder, as a web-based tool, can find the significant GO terms among plenty of genes and it is publicly available at http://go.princeton.edu/cgi-bin/GOTermFinder [29]. Therefore, GOTermFinder offers some significant information for the biological explanation of high-throughput experiments. The threshold parameters are given as follows: maximum P-value is set to 0.01 and minimum number of gene products is set to 2. In the following, only the primary outcomes of GO Term Enrichment are shown.

(b) Term responding to stress

The numbers of genes and P-value of response to stress (GO:0006950) in root and shoot samples are given in Table 3.

**Table 3:** Response to stress (GO:0006950). In this table, the response to stress on differentially expressed genes is shown, whose background frequency in TAIR is 4044/30322 (13.3%), where 4044/30322 represents having 4044 genes response to stimulus in whole 30322 genes. SF and PV represent the sample frequency and P-value, respectively. The sample frequency, e.g. 223, represents the method identifies 500 genes, in which there are 223 genes responding to stress. Root and shoot denote the root samples and shoot samples, respectively.

Stress Type		SPCA		PMD		CIPMD		SVM-RFE		PRFE	
		SF	PV	SF	PV	SF	PV	SF	PV	SF	PV
Cold	root	223	1.66E-64	233	9.92E-72	264	7.64E-98	218	7.14E-61	245	6.05E-81
		44.8%		46.6%		52.9%		43.9%		49.0%	
Cold	shoot	219	1.47E-61	213	4.44E-57	243	6.84E-80	204	1.07E-50	221	6.36E-63
		44.0%		42.7%		48.7%		40.9%		44.5%	
Drought	root	231	3.60E-70	222	2.27E-63	279	2.50E-111	225	7.69E-66	232	1.36E-70
		46.2%		44.4%		55.8%		45.2%		46.4%	
Drought	shoot	198	5.05E-47	246	2.47E-82	255	5.89E-90	201	1.02E-48	277	5.61E-109
		39.8%		49.3%		51.1%		40.3%		55.4%	
Heat	root	152	5.73E-21	169	1.39E-29	277	1.03E-109	242	1.11E-78	180	8.81E-36
		30.5%		33.9%		55.5%		48.4%		36.2%	
Heat	shoot	187	4.49E-40	174	3.51E-32	264	1.51E-97	225	1.21E-65	213	6.55E-57
		37.6%		34.8%		52.8%		45.1%		42.8%	
Osmotic	root	172	4.39E-31	160	8.07E-25	234	1.78E-72	227	6.15E-67	176	4.04E-33
		34.4%		32.0%		46.8%		45.4%		35.2%	
Osmotic	shoot	192	4.96E-43	227	4.12E-67	246	2.30E-82	183	2.88E-37	226	5.21E-66
		38.5%		45.4%		49.3%		36.6%		45.2%	
Salt	root	178	1.79E-34	246	3.88E-82	232	5.58E-71	218	1.76E-60	243	2.57E-79
		35.6%		49.2%		46.4%		43.7%		48.6%	
Salt	shoot	169	1.85E-29	176	1.34E-33	236	2.90E-74	202	3.32E-49	181	2.16E-36
		33.8%		35.3%		47.3%		40.4%		36.4%	
UV-B	root	153	2.26E-21	165	2.34E-27	262	9.89E-96	222	2.04E-63	178	2.35E-34
		30.6%		33.0%		52.4%		44.5%		35.7%	
UV-B	shoot	249	4.18E-85	295	3.30E-127	277	1.06E-109	186	4.81E-39	300	4.38E-132
		50.0%		59.1%		55.5%		37.2%		60.2%	

From Table 3 we can see that all the five methods can identify the differentially expressed genes with higher sample frequency which can reflect the accuracy of the feature extraction method and lower P-value. PRFE, SPCA and PMD are unsupervised methods, so we first compare the three algorithms. In the 12 terms, there is only two of them (osmotic stress in shoot samples and salt stress in root samples) that the proposed method is surpassed by PMD slightly. In the remaining 10 terms, PRFE method outperforms PMD and SPCA. Generally speaking, since supervised methods take the class labels into consideration, they usually have better performance than unsupervised methods. However, unsupervised methods have unique advantages than supervised methods. For example, when a data set has no class information, in this case the supervised methods are always helpless in analyzing the data set, but unsupervised methods like PMD, SPCA and PRFE can analyze the data without class labels effectively. Table 3 shows that PRFE outperforms CIPMD on drought stress in shoot samples, salt stress in root samples and UV-B stress in shoot samples. Furthermore, only on drought stress in shoot and root samples, osmotic stress in root samples, salt stress in shoot samples and UV-B stress in root samples SVM-RFE is superior to our method.

(c) Term responding to abiotic stimulus

Table 4 shows the gene numbers and P-value of response to abiotic stimulus (GO:0009628) in root and shoot samples.

**Table 4:** Response to abiotic stimulus (GO:0009628). In this table, the response to abiotic stimulus on differentially expressed genes is shown, whose background frequency in TAIR is 2842/30322 (9.4%), where 2842/30322 represents having 2842 genes response to stimulus in whole 30322 genes. SF and PV represent the sample frequency and P-value, respectively. The sample frequency can reflect the identify accuracy of the diffenrent methods, e.g. 155, represents the method identifies 500 genes, in which there are 155 genes responding to abiotic stimulus. Root and shoot denote the root samples and shoot samples, respectively.

Stress Type		SPCA		PMD		CIPMD		SVM-RFE		PRFE	
		SF	PV	SF	PV	SF	PV	SF	PV	SF	PV
Cold	root	155	3.31E-40	168	6.34E-49	172	8.99E-52	180	4.29E-58	178	5.02E-56
		31.1%		33.6%		34.4%		36.2%		35.6%	
Cold	shoot	148	1.13E-35	179	3.57E-57	180	6.31E-58	178	2.93E-56	184	4.24E-61
		29.7%		35.9%		36.1%		35.7%		37.0%	
Drought	root	134	4.66E-27	118	1.51E-18	170	2.28E-50	185	8.52E-62	136	4.85E-28
		26.8%		23.6%		34.0%		37.1%		27.2%	
Drought	shoot	126	8.27E-23	164	3.49E-46	177	1.21E-55	177	1.58E-55	183	8.05E-60
		25.3%		32.9%		35.5%		35.5%		36.6%	
Heat	root	108	6.69E-14	141	3.11E-31	173	1.13E-52	198	5.99E-72	148	1.37E-35
		21.6%		28.3%		34.7%		39.6%		29.8%	
Heat	shoot	142	6.07E-32	148	2.04E-35	173	1.64E-52	192	3.28E-67	169	1.18E-49
		28.5%		29.6%		34.6%		38.5%		33.9%	
Osmotic	root	132	6.69E-26	120	1.42E-19	165	4.88E-47	193	7.66E-68	136	4.76E-28
		26.4%		24.0%		33.1%		38.6%		27.2%	
Osmotic	shoot	146	2.65E-34	171	4.55E-51	166	1.28E-47	186	2.77E-62	176	1.67E-54
		29.3%		34.2%		33.3%		37.2%		35.2%	
Salt	root	119	4.82E-19	152	5.13E-38	161	5.65E-44	183	4.41E-60	114	1.00E-39
		23.8%		30.4%		32.2%		36.7%		22.8%	
Salt	shoot	145	1.45E-33	148	1.12E-35	179	5.52E-57	183	6.12E-60	153	7.9E-39
		29.0%		29.7%		35.8%		36.6%		30.8%	
UV-B	root	101	6.70E-11	120	1.49E-19	176	7.04E-55	184	7.27E-61	135	1.53E-27
		20.2%		24.0%		35.3%		36.9%		27.1%	
UV-B	shoot	154	1.49E-39	153	8.81E-39	184	5.20E-61	179	7.26E-57	171	4.3E-51
		30.9%		30.7%		36.9%		35.8%		34.3%	

As Table 4 lists, each of the five methods can acquire good performance when it is used to identify the differentially expressed genes responding to abiotic stimulus. We still analysis the unsupervised methods at first. The proposed method is superior to SPCA and PMD in 11 terms, only for the salt stress data set in root samples, PRFE method is dominated by PMD and SPCA. For the supervised methods, PRFE is superior to CIPMD on cold stress in the root and shoot samples, drought stress in the shoot samples and osmotic stress in the shoot samples. On cold stress in the shoot samples and drought stress in the shoot samples our method outperforms SVM-RFE.

#### 3.2.3 Results on leukemia data

(a) Data source and GO analysis

The leukemia data set consists of 27 cases of acute lymphoblastic leukemia (ALL) and 11 cases of acute myelogenous leukemia (AML) [27]. It is summarized by a 5000×38 matrix for further processed.

For fair comparison, 100 genes are identified by the five methods. The Gene Ontology (GO) enrichment of functional annotation of the identified genes by five methods is detected by ToppFun [30] which is publicly available at http://toppgene.cchmc.org/enrichment.jsp. Here, GO: Biological Process is the main objective to analysis. The P-value is set to 0.01 and number of gene limits is set to 2 by ToppFun.

(b) Terms relate to leukemia data

Table 5 lists the top 10 closely related terms corresponding to different methods. From Table 5 it can be clearly found that PRFE method outperforms PMD and CIPMD in all 10 terms. Our method can identify the same number of genes as SPCA in the following three terms: defense response, regulation of immune system process and leukocyte activation. However, we have lower P-values than SPCA in these three terms. Though in the term: cell activation our method is surpassed by SPCA, PRFE outperforms SPCA in the remaining terms. SVM-RFE method performs best in all the five methods. But in the term: response to reactive oxygen species, only PRFE and PMD can identify differentially expressed genes, in addition, PRFE can identify more genes than PMD.

**Table 5:** The terms of genes identified by different methods. In this table, 'Term in Genome' denotes the number of genes associated with the term in global genome; 'Input' denotes the number of genes associated with the term from input.

Rank	Name	SPCA	PMD	CIPMD	SVM-RFE	PRFE	Term in Genome
		Input PV	Input PV	Input PV	Input PV	Input PV	
1	immune response	29	27	27	36	33	1416
		5.39E-14	2.04E-12	2.92E-12	4.10E-20	3.31E-18	
2	defense response	30	26	24	34	30	1515
		4.02E-14	6.40E-11	3.04E-9	3.43E-17	1.69E-14	
3	response to biotic stimulus	19	15	15	24	22	760
		1.22E-10	2.69E-7	3.24E-7	3.70E-15	8.46E-14	
4	response to other organism	19	14	15	24	21	726
		5.60E-11	9.42E-7	1.80E-7	1.34E-15	3.58E-13	
5	response to external biotic stimulus	19	14	15	24	21	726
		5.60E-11	9.42E-7	1.80E-7	1.34E-15	3.58E-13	
6	response to reactive oxygen species	None	8	None	None	11	170
		None	3.91E-7	None	None-	6.26E-11	
7	regulation of immune system process	23	14	14	25	23	1212
		2.19E-10	9.56E-7	3.53E-6	1.21E-11	1.19E-10	
8	leukocyte activation	18	17	17	22	18	695
		2.33E-10	1.53E-9	1.91E-9	6.36E-14	1.44E-10	
9	hematopoietic or lymphoid organ development	18	14	None	19	19	795
		2.00E-9	2.74E-6	None	5.41E-10	1.57E-10	
10	cell activation	22	19	19	26	20	916
		6.47E-12	2.16E-9	2.76E-9	2.48E-15	2.31E-10	

**Fig 5:**
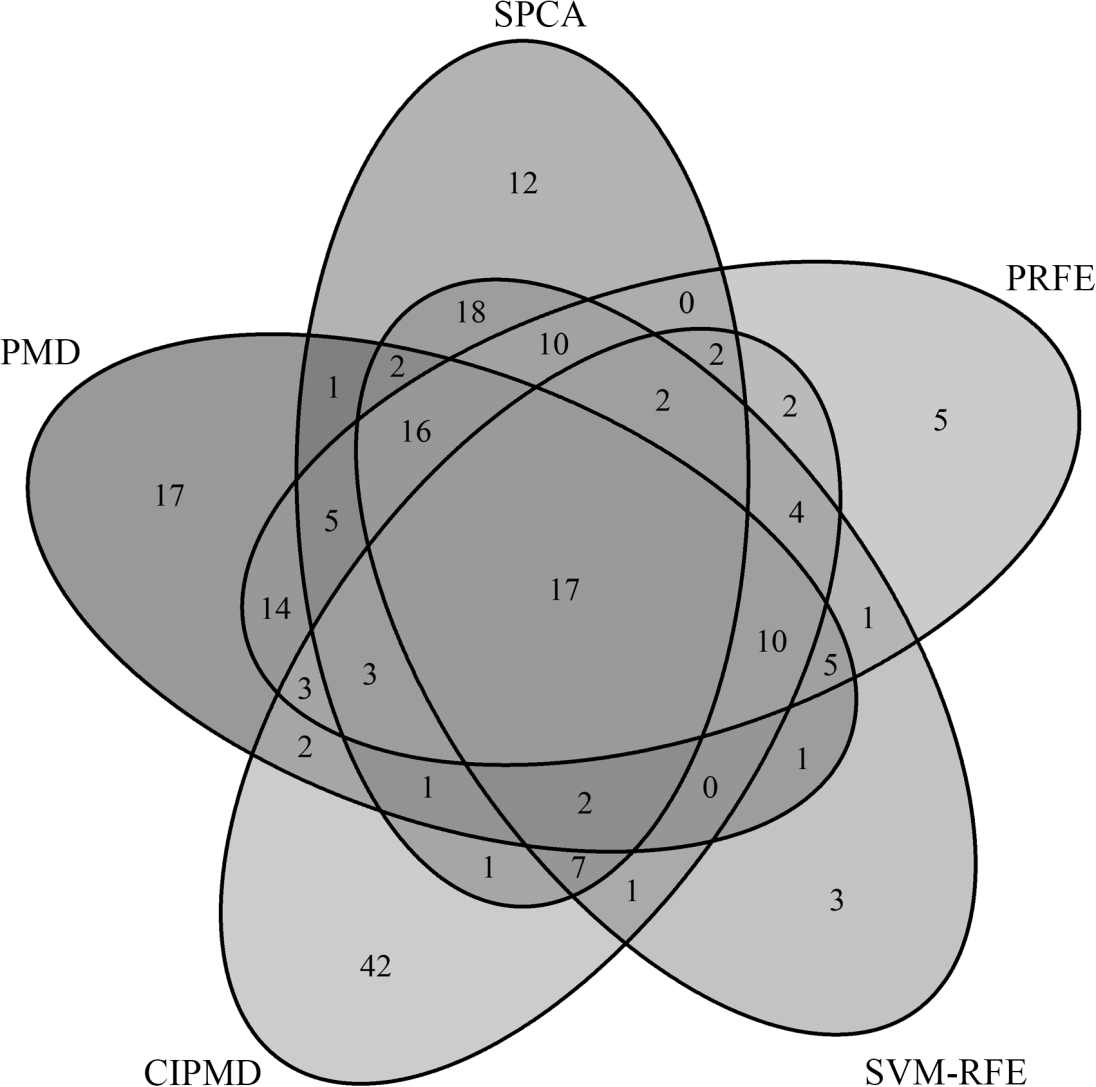
Venn diagram of five methods on leukemia data.

To further study the performance of the methods, a Venn diagram is shown in Fig 5. From Fig 5 we can see that both PRFE and SVM-RFE identify less 'unique' differentially expressed genes than PMD, SPCA and CIPMD. There are 17 genes shared by all the five methods. The detailed information of the 5 'unique' differentially expressed genes extracted by PRFE are shown in Table 6. From Table 6 we can see that the 5 'unique' differentially expressed genes extracted by PRFE and neglected by other methods are associated with leukemia. Therefore, this suggests that PRFE is an excellent method for identifying differentially expressed genes on leukemia data set.

**Table 6:** The detailed information of the 5 'unique' genes identified by PRFE.

NO.	Affymetrix ID	Gene Symbol	Function of Genes
1	S53911_at	CD34	The protein encoded by this gene may play a role in the attachment of stem cells to the bone marrow extracellular matrix or to stromal cells.
2	AFFX-M27830_5_at		GB virus C effect on hepatitis C virus (HCV)/human immunodeficiency virus (HIV) co-infected patients: liver.
3	M21624_at	TRAJ17	T cell receptor alpha joining 17.
4	X60486_at	HIST1H4C	Histones are basic nuclear proteins that are responsible for the nucleosome structure of the chromosomal fiber in eukaryotes.
5	M57466_s_at	HLA-DPB1	HLA-DPB belongs to the HLA class II beta chain paralogues. This class II molecule is a heterodimer consisting of an alpha (DPA) and a beta chain (DPB), both anchored in the membrane. It plays a central role in the immune system by presenting peptides derived from extracellular proteins.

(c) Genes correlate with leukemia data

To further study the correlation between the identified genes and leukemia data, they are verified based on the literatures. For simplicity, the top 30 genes identified by PRFE are taken into consideration. Depending on [31], there are 50 genes most closely correlated with the leukemia data set distinction in the known samples. Among these 50 genes, 3 genes are contained in the top 30 genes identified by PRFE. The Affymetrix ID and Gene Symbol of 3 genes are given as follows: M13792_at (ADA), M69043_at (NFKBIA), Y00787_s_at (IL8). The article [31] was written by Golub et al. in 1999, at that time, only 50 genes were found to be associated with the leukemia data set. As time goes on, many other genes were found to be closely correlated with leukemia. According to [32], there are 210 genes is related to leukemia. All the 30 genes identified by our method can be found in [32]. The detailed information of the 30 genes are shown in Table 7.

**Table 7:** The detailed information of the 30 genes identified by PRFE.

NO.	Affymetrix ID	Gene Symbol	Function of Genes
1	M25079_s_at	HBD	The delta (HBD) and beta (HBB) genes are normally expressed in the adult: two alpha chains plus two beta chains constitute HbA, which in normal adult life comprises about 97% of the total hemoglobin.
2	X57351_s_at	IFITM2	Interferon induced transmembrane protein 2.
3	X00274_at	HLA-DRA	HLA-DRA is one of the HLA class II alpha chain paralogues. This class II molecule is a heterodimer consisting of an alpha and a beta chain, both anchored in the membrane. It plays a central role in the immune system by presenting peptides derived from extracellular proteins.
4	Z84721_cds2_at	HBA2	The human alpha globin gene cluster located on chromosome 16 spans about 30 kb and includes seven loci: 5'- zeta-pseudozeta-mu-pseudoalpha-1-alpha-2 (HBA2)- alpha-1-theta-3'.
5	X00437_s_at	TRBC1	T cell receptor beta constant 1.
6	D64142_at	H1FX	H1 histone family, member X. Histones are basic nuclear proteins that are responsible for the nucleosome structure of the chromosomal fiber in eukaryotes.
7	M11147_at	FTL	This gene encodes the light subunit of the ferritin protein. Ferritin is the major intracellular iron storage protein in prokaryotes and eukaryotes.
8	M13560_s_at	CD74	The protein encoded by this gene associates with class II major histocompatibility complex (MHC) and is an important chaperone that regulates antigen presentation for immune response. It also serves as cell surface receptor for the cytokine macrophage migration inhibitory factor (MIF) which, when bound to the encoded protein, initiates survival pathways and cell proliferation.
9	Y00433_at	GPX1	This gene encodes a member of the glutathione peroxidase family. Glutathione peroxidase functions in the detoxification of hydrogen peroxide, and is one of the most important antioxidant enzymes in humans.
10	V00594_s_at	MT2A	Metallothionein 2A.
11	L19779_at	HIST2H2AA4	Histone cluster 2, H2aa4. Histones are basic nuclear proteins that are responsible for the nucleosome structure of the chromosomal fiber in eukaryotes.
12	AFFX-HUMRGE/M10098_5_at	SRP68	This gene encodes a subunit of the signal recognition particle (SRP). The SRP is a ribonucleoprotein complex that transports secreted and membrane proteins to the endoplasmic reticulum for processing.
13	AFFX-HUMRGE/M10098_3_at	SRP68	This gene encodes a subunit of the signal recognition particle (SRP). The SRP is a ribonucleoprotein complex that transports secreted and membrane proteins to the endoplasmic reticulum for processing.
14	M91036_rna1_at	HBG2	The gamma globin genes (HBG1 and HBG2) are normally expressed in the fetal liver, spleen and bone marrow.
15	M12886_at	IL23A	This gene encodes a subunit of the heterodimeric cytokine interleukin 23 (IL23). IL23 is composed of this protein and the p40 subunit of interleukin 12 (IL12B).
16	X82240_rna1_at	TCL1A	Overexpression of the TCL1 gene in humans has been implicated in the development of mature T cell leukemia.
17	M16279_at	CD99	The protein encoded by this gene is a cell surface glycoprotein involved in leukocyte migration, T-cell adhesion, ganglioside GM1 and transmembrane protein transport, and T-cell death by a caspase-independent pathway.
18	M13792_at	ADA	This gene encodes an enzyme that catalyzes the hydrolysis of adenosine to inosine. Various mutations have been described for this gene and have been linked to human diseases.
19	M33600_f_at	HLA-DRB1	HLA-DRB1 belongs to the HLA class II beta chain paralogs. The class II molecule is a heterodimer consisting of an alpha (DRA) and a beta chain (DRB), both anchored in the membrane. It plays a central role in the immune system by presenting peptides derived from extracellular proteins.
20	M21186_at	CYBA	Cytochrome b is comprised of a light chain (alpha) and a heavy chain (beta). This gene encodes the light, alpha subunit which has been proposed as a primary component of the microbicidal oxidase system of phagocytes.
21	L06797_s_at	CXCR4	This gene encodes a CXC chemokine receptor specific for stromal cell-derived factor-1. The protein has 7 transmembrane regions and is located on the cell surface.
22	X68277_at	DUSP1	The expression of DUSP1 gene is induced in human skin fibroblasts by oxidative/heat stress and growth factors. It specifies a protein with structural features similar to members of the non-receptor-type protein-tyrosine phosphatase family, and which has significant amino-acid sequence similarity to a Tyr/Ser-protein phosphatase encoded by the late gene H1 of vaccinia virus.
23	M69043_at	NFKBIA	This gene encodes a member of the NF-kappa-B inhibitor family, which contain multiple ankrin repeat domains. The encoded protein interacts with REL dimers to inhibit NF-kappa-B/REL complexes which are involved in inflammatory responses.
24	X58529_at	IGHM	Immunoglobulin heavy constant mu. Immunoglobulins (Ig) are the antigen recognition molecules of B cells.
25	J04456_at	LGALS1	The galectins are a family of beta-galactoside-binding proteins implicated in modulating cell-cell and cell-matrix interactions. This gene product may act as an autocrine negative growth factor that regulates cell proliferation.
26	X78992_at	ZFP36L2	This gene is a member of the TIS11 family of early response genes. Family members are induced by various agonists such as the phorbol ester TPA and the polypeptide mitogen EGF.
27	X12671_rna1_at	HNRNPA1	The protein encoded by this gene has two repeats of quasi-RRM domains that bind to RNAs. It is one of the most abundant core proteins of hnRNP complexes and it is localized to the nucleoplasm.
28	M33680_at	CD81	The protein encoded by this gene is a member of the transmembrane 4 superfamily, also known as the tetraspanin family. Most of these members are cell-surface proteins that are characterized by the presence of four hydrophobic domains.
29	Y00787_s_at	IL8	Gene expression profiling study of contribution of GM-CSF and IL-8 to the CD44-induced differentiation of acute monoblastic leukemia.
30	S73591_at	TXNIP	Thioredoxin interacting protein.

## Conclusion

In this paper, based on the Schatten *p*-norm and *L*_*p*_-norm, we propose a novel feature extraction method named as PRFE to identify differentially expressed genes in gene expression data sets. The method combined the Schatten *p*-norm and *L*_*p*_-norm to provide an effective way for gene identification. Numerous experiments on simulation data and real gene expression data sets demonstrate that the proposed method has a better performance than the other state-of-the-art gene identification methods. Moreover, the identified genes are confirmed that they are closely correlated with the corresponding gene expression data.
